# Research protocol: a realist synthesis of contestability in community-based mental health markets

**DOI:** 10.1186/s13643-015-0025-3

**Published:** 2015-03-25

**Authors:** Jo Durham, Amara Bains

**Affiliations:** Faculty of Medicine & Biomedical Sciences, The University of Queensland, School of Public Health, Herston, Brisbane, Queensland 4006 Australia; Queensland Alliance for Mental Health, 1/78 Logan Road, Woolloongabba, Brisbane, Queensland 4102 Australia

**Keywords:** Realist synthesis, Realist review, Contestable markets, Contestability, Community-based organisations, Mental health, Community mental health

## Abstract

**Background:**

In most developed nations, there has been a shift from public services to a marketisation of public goods and services - representing a significant reform process aiming to transform the way in which community-based human services, such as health, are delivered and consumed. For services, this means developing the capacity to adapt and innovate in response to changing circumstances to achieve quality. The availability of rigorous research to demonstrate whether a market approach and contestability, in particular, is a coherent reform process is largely absent. Contestability operates on the premise that better procurement processes allow more providers to enter the market and compete for contracts. This is expected to create stimulus for greater efficiencies, innovation and improved service delivery to consumers. There is limited understanding, however, about how community-based providers morph and re-configure in response to the opportunities posed by contestability. This study focuses on the effect of a contestability policy on the community-managed mental health sector.

**Methods/design:**

A realist review will be undertaken to understand how and why the introduction of contestability into a previously incontestable market influences the ways in which community-based mental health providers respond to contestability. The review will investigate those circumstances that shape organisational response and generate outcomes through activating mechanisms. An early scoping has helped to formulate the initial program theory. A realist synthesis will be undertaken to identify relevant journal articles and grey literature. Data will be extracted in relation to the emerging contextual factors, mechanisms and outcomes and their configurations. The analysis will seek patterns and regularities in these configurations across the extracted data and will focus on addressing our theory-based questions.

**Discussion:**

Increasingly, community-based mental health markets are moving to contestability models. Rigorous research is needed to understand how such markets work and in what contexts. The knowledge gained from this study in community-based mental health will provide valuable lessons in how contestability works, in what circumstances and who benefits when. The results of the proposed research will be useful to policy-makers and may be applicable in other contexts beyond the community-based mental health sector.

**Systematic review registration:**

PROSPERO CRD42015016808

## Background

Over the last three decades, there has been a general shift from public services to a marketisation of public goods and services [1-8]. A market is the organisation through which the consumption and production of goods and services takes place through voluntary exchanges [9-13]. The shift to a market approach in the delivery of public services is underpinned by the belief that market mechanisms allocate resources efficiently and effectively [8,10,14]. More recently, the concept of maximising consumer choice has also been used to justify employing market mechanisms in government-funded human services [1,2,8]. In many high-income countries, public utilities and other previously nationalised industries have been subject to competitive markets through privatisation, for example, water and electricity [1]. Increasingly, however, other not-for-profit providers, notably health service and the community-based sector, are also having to operate in contestable markets [1]. These competitive tendering processes assume that competition between for- and not-for-profit providers act as a catalyst to improve service delivery efficiency, through business process improvement [1,14].

A perfectly contestable market is one where both entry and exit for suppliers is costless and where potential new suppliers have access to all production techniques available to the incumbents and are able to attract the incumbents’ customers [1,2,8]. The premise is that contestability allows more providers to enter the market and compete for contracts, creating the stimulus for greater efficiencies, innovation and improved service delivery to consumers. [1,2,14]. According to market theory, when operating under conditions of perfect competition and perfect information, rational market players respond to price signals, and this price-regulated, money-based exchange ensures efficiency and equilibrium between supply and demand and optimum allocation of resources [10,11,13,15]. While perfect contestability may be rare, a market is generally considered ‘contestable’ if there are no insurmountable barriers to preclude at least one new supplier competing for the consumers of another supplier [1,14].

Contestable markets work by combining numerous and varying supply and demand components and adapting and responding to context. Feedback in contestable markets is iterative, and how markets work depends on human behaviour [16-19]. In this sense, introducing contestability to the provision of public goods is a complex policy intervention. This is because the policy relies in the behaviour of people, who may have different understandings of the policy, and as with any intervention, it is embedded in context and time. Other aspects of complex interventions that are also relevant to contestability are that the policy is likely to have multiple, contested outcomes, will be implemented alongside other interventions and will have long chains of implementation, for example, from the health department down to the patient [20]. Further, as with other complex interventions, outcomes are emergent and change over time as the intervention becomes part of the context [20].

In Australia, government-funded community services are shifting to contestable markets, and in Queensland, the mental health sector is being particularly affected. Research in Australia on the commercialisation of public utilities suggests that there have been some improvements in terms of efficiency, client satisfaction and accountability [21]. Other organisational responses were reported to include downsizing and a clearer focus on costs and return [21]. Contextual factors include the capacity of the government departments to manage contracts and shape the market to achieve effective and efficient outcomes for clients [8,21]. In the community-based organisation (CBO) not-for-profit sector, however, there is limited evidence that explains how and in what circumstances CBOs adapt in response to this policy reform [1,2] and whether CBOs’ behaviour and practices are similar to for-profit organisations [22].

Understanding how CBOs adapt to contestable markets is an important step in enabling policy-makers to introduce and manage contestable markets to promote efficient, effective and equitable services. In this research, we propose to begin to address this gap through a realist synthesis of the literature. The main question we seek to answer is as follows: why, how and in what context do CBOs providing mental health services respond to the introduction of contestability? This is not to evade the important issue of how and why these strategies affect services but is an initial step in understanding the implications of contestability in community-based mental health markets. Using a realist approach [12,23], our aim is to provide policy guidance to government and mental health CBOs on the management of the transition to a contestable market.

## Methods/design

We have chosen a realist approach for our research [24,25] as it is ideal for evaluating complex interventions because it allows a focus on actual outcomes in ‘real world’ settings as well as on the contextual factors that influence outcomes [26]. It is a theoretically driven, interpretive approach to synthesising qualitative, quantitative and mixed-methods research evidence [12,23-25,27-29]. The use of theory allows a better understanding of how policy is supposed to work and helps to capture the complexity of interventions by including context in the analysis [26,30].

### The realist approach

The realist approach assumes that an intervention does not work in itself. From a realist perspective, it is the mechanisms underlying the intervention that act (or otherwise) to generate outcomes [20,31,32]. These mechanisms are influenced by the context in which the intervention is implemented. The intent of a realist review is to develop middle-range theories that explain how the context (C) influences mechanisms (M) to generate outcomes (O), often called context-mechanism-outcome (C-M-O) configurations [23]. The approach is underpinned by a realist perspective of social change whereby social phenomena are constructed by the actions of individuals and by their understanding of such phenomena [26,29,33,34]. The way individuals act, however, is also constrained by social structures, and it is important to understand how individuals and the structure interact to produce outcomes [16,17,33]. In the realist approach, mechanisms are elements from the reasoning of actors involved in interventions such as beliefs, values, desires and cognitive processes that influence behavioural choices [20,27]. Context includes social, cultural, historical or institutional factors that enable or constrain actors [11,13,17]. Outcomes are the expected or unexpected intermediate and final outcomes. A realist approach first articulates the underlying program theory of a policy or intervention. It then empirically tests this theory to investigate whether, why or how, policies or programs cause observed outcomes, for whom and in what circumstances.

In the present study, identifying the mechanisms will assist us to explain how mental health CBOs react to contestability. Working from a realist perspective, it is assumed that there are several C-M-O configurations within the program theory, in this case, the program theory describing the way in which CBOs respond to the introduction of contestability. The preliminary review of the literature will enable us to draft initial C-M-O configurations which will be refined through our realist synthesis [25]. Identifying transferable mechanisms will permit the research to extend to a level of abstraction potentially making the theory or theories developed in the community-managed mental health sector applicable in other contexts [26,28].

### Research aim and objectives

The primary aim of this synthesis is to explain why, how and in what contexts mental health CBOs transition to a contestable market as well as identify the underlying theories that explain outcomes for CBOs. Our overall objective is to refine our program theory by developing a set of C-M-O statements on the contexts in which particular mechanisms generate particular outcomes in order to assist policy-makers in shaping contestable markets to deliver efficient services. Our specific objectives are as follows:

1. Document the strategies CBOs in community mental health services use to respond to contestability.
2. Examine the outcomes (positive, for example, the desired outcomes of equitable and efficient community-based mental health care and negative and/or unintended outcomes) and the intermediate outcomes of the strategies CBOs employ.
3. Identify the mechanisms that generate these different outcomes (positive and negative).
4. Investigate the circumstances in which these different outcomes are generated.
5. Develop a realist program theory that synthesises review findings of how community-based organisations in community mental health respond to contestability and the outcomes for those organisations.

The review will follow Pawson’s five stages in conducting realist reviews [23]. While we present the methods in five steps, the review will be iterative with the researchers often moving between the different steps [25,35].

### Step 1: identifying potential theories

An initial scoping review of the literature uncovered a number of key elements through which contestable markets are expected to work. We have done this by reviewing key government policy documents and consulting key stakeholders in an expert reference group that includes policy-makers, public servants responsible for overseeing the implementation of the contestability policy, CBO program managers and academics. An expert reference group will assist in identification of additional articles and documents for inclusion in the review as well as provide a forum to test our emerging understandings of the program theory.

### Step 2: search strategy

Following our initial scoping of policy and discussions with our reference group, we will undertake a realist review to map the elements of contestable markets with a view to further uncovering the underlying theory of contestability in CBO markets. This search will be undertaken using the databases Ovid Medline, Embase, Web of Knowledge, EconLit and Web of Science. Combinations of key words in English and their truncations will be entered in these databases; the relevance of the retrieved documents will be assessed according to exclusion and inclusion criteria and how each study clarifies the C-M-O configurations. Bibliographic references from the included documents will be reviewed using the snowballing technique to identify additional documents. Given that grey literature is a relevant source of information for realist reviews, evaluation reports or policy documents published by governments, international organisations, non-governmental organisations and consultancy firms, as well as dissertations and theses, may also be included [25]. The search for evidence will be driven by our research objectives and will use an iterative process of extending and refocusing the review depending on the identified sources as the review and our understanding of the program theory develop [20,25,35]. Searching will stop when saturation is reached and there is sufficient evidence to reasonably claim that the (revised) theory is plausible [30]. Table 1 provides the initial word search strategy. References will be compiled in EndNote.

**Table 1:** **Initial search terms**

**Community-based services**	**Public sector reform**	**System transformation**
Community mental health	Contestable	Organisational innovation
NGOs	Public health economy	Transformation processes
Not-for-profit	Health care reform	Organisation change
	Mental health care reform	Organisation capacity
	Policy making	Organisation resilience
	Health market	Learning organisations
		Capitals/assets
		System change
		System transformation
		Complex systems
		Adaptive capacity

### Step 3: study selection criteria and procedures

Documents will be included in the review based on relevance, that is, the extent to which they can provide data to inform program theory development and clarify the C-M-O configurations [30]. Documents may include editorials, opinion pieces, commentaries, evaluations and reviews, and the unit of analysis will be the aspects of the document that relate to the relationships between context, mechanism and outcomes and how they contribute to our understanding of how contestable markets work for CBOs. The inclusion criteria will be papers related to community-based mental health care target group, in any country and published between January 2000 and January 2015. The inclusion criteria will also be guided by the research objectives and will include screening title, abstracts and keywords of documents identified in the initial search. Based on discussion between the two reviewers (JD and AB), the inclusion criteria will be refined and may, for example, include extending the inclusion criteria to papers in community-based health care if insufficient papers are found that relate to community-based mental health services.

Following Brennan and colleagues [35], a random sample of 10% of articles will be assessed and discussed by the two authors and the remainder completed by one reviewer although it is expected that there will some papers that will require discussion between both reviewers to decide whether the paper should be included in the review [35].

### Step 4: data extraction

The documents included in the review will initially be compiled in Excel with data extracted based on how, why and in what contexts contestable markets are assumed to work in the context of CBOs. During the data extraction process, aspects of each paper will be assessed for relevance guided by the following questions which will be used to assist us in selecting documents for the review:

1. Does the paper (or an aspect of it) describe a contestable market?
2. Is the paper (or an aspect of it) implicitly or explicitly underpinned by the theory of contestable markets?
3. Does the paper (or an aspect of it) provide details about the outcomes of contestable markets for community-based organisations and/or their clients?
4. Does the paper (or an aspect of it) provide information on the context?
5. Does the paper (or an aspect of it) provide some evidence that will contribute to the synthesis and our emerging theory?

Realist reviews do not rely only on quantitative data and analytic techniques [23,30], and in this review, it is expected that most of the data will be qualitative. This data will be entered in to NVivo software package for coding. Where quantitative data is available, it will be analysed using appropriate analytic techniques and software, for example, SPSS. Our initial theory of how these markets are expected to work will be used to analyse the data and will be further specified in an iterative manner allowing integration of new explanatory elements of our emerging theory. Assessing the quality of papers will be done using the Mixed Methods Appraisal Tool [36]. This tool has been chosen because it allows an assessment of quantitative and qualitative data and provides one tool for concomitantly appraising diverse study designs [36]. It has theoretical and content validity and has been tested for efficiency and reliability [36,37]. The tool will not be applied to the whole study but only to the pertinent aspects of each study, that is, only those aspects that relate to our program theory [32]. To ensure a transparent process, a summary table will be developed specifying the authors, objectives, type of study, different methodological aspects and study country.

### Step 5: data synthesis

Using a mix of inductive and deductive analytical processes, each paper will be examined for evidence based on how it supports, refutes, reinterprets or refocuses our initial theory. Deductive codes will be developed in advance based on our initial program theory. As we analyse the data, additional codes will be created for sections of text that seem relevant to the program theory. During this coding process, we will seek to determine if the coded extract refers to context, mechanism or outcome, what the C-M-O configuration might be and how it contributes to our program theory. We anticipate that evidence of outcomes from some papers may allow insights about context in another. Where outcomes are different in certain contexts we will seek to explain how and why these outcomes occur differently [20,35]. Each study will contribute to clarifying or reformulating C-M-O configurations [26,38]. Papers may be grouped by similar propositional statements in order to further identify patterns and identify similar contexts and mechanisms.

### Validity

The iterative process of understanding how contestable community-based mental health markets works will require the researchers to move between empirical data and construction of C-M-O configurations [24]. This process, alongside our deliberate inclusion of context in the analysis will enhance internal validity and the generalisation potential of the identified mechanisms [24,32]. The use of an expert reference group to provide feedback, help identify additional studies and review the findings also provide additional safeguards to validity [39].

### Ethics

As the study does not involve primary research, it does not require formal ethical approval. The study will, however, follow the ethical standards of utility, usefulness, feasibility, propriety, accuracy and accountability [33].

### Dissemination

The research approach will allow us to draw on the existing body of evidence to produce an explanatory understanding of how contestable markets work for community-based mental health and provide insights into which populations benefit, in what ways and in what circumstances. We will use the publication standards for realist synthesis for this review [32]. The results of the proposed research will be useful to policy-makers, CBO managers and academics in understanding how to effectively manage CBO mental health markets. The results will be published in a peer-reviewed journal that focusses on community-based health care and policy. In addition, the research will be disseminated through the expert reference group and our local and professional networks including health care managers and administrators, community-based organisations and policy-makers seeking to understand how contestability can be facilitated to enhance health services. The dissemination strategy for this audience will be through meetings and policy briefs. Together, these strategies will allow for broad dissemination and discussion of our findings to policy-makers and practitioners.

## Discussion

Increasingly, health markets are moving to contestability models. Rigorous research is needed to understand how such markets work and in what contexts. The knowledge gained from this study of community-based mental health markets will provide valuable transferrable lessons in how contestability works, in what circumstances and who benefits, when. By capturing the relationship between context and process in transforming the way in which CBOs providing mental health services work and respond to contestability, the findings of this review will be particularly pertinent for policy-makers in furthering debate on how to provide universal access to sustainable community-based mental health care. It will also identify potentially transferable mechanisms that can be tested with other CBOs making such a shift.

As with all research methods, realist reviews have some limitations. For example, compared to systematic reviews, realist reviews are harder to reproduce as selecting papers and identifying candidate theories requires judgment that is often based on a mixture of experience, intuition and prudence to identify those with greatest relevance [23,30]. To minimise this limitation, we have built in a number of safeguards including developing a summary table and methodological details of papers included in the review. This will ensure we have sufficient transparency to enable others to see how we arrived at our findings and conclusions. Similar to other reviews, however, the quality is, to an extent, reliant on the transparency and adequacy of reporting by original authors; therefore, where possible, we will contact authors for clarification. In addition, our emerging findings will continually be checked for plausibility with our reference group [39]. Finally, our interest is how and why and in what context do CBOs providing mental health services choose to respond to the introduction of contestability. This is not to sidestep the questions of how and why these strategies work for clients. We believe, however, this focus on CBO response is a required initial step in understanding the implications of contestability on the client’s experience and treatment outcomes. By identifying the mechanisms and contexts in place for described outcomes, both policy-makers and community organisations can co-design service provision for greater efficiencies and equity.

## Abbreviations

CBO: community-based organisation
C-M-O: context, mechanism, outcome
